# The Immune System's Role in the Consequences of Mild Traumatic Brain Injury (Concussion)

**DOI:** 10.3389/fimmu.2021.620698

**Published:** 2021-02-15

**Authors:** Laura N. Verboon, Hiren C. Patel, Andrew D. Greenhalgh

**Affiliations:** ^1^Division of Infection, Immunity and Respiratory Medicine, Faculty of Biology, Medicine and Health, School of Biological Sciences, The University of Manchester, Manchester, United Kingdom; ^2^Division of Cardiovascular Sciences, Salford Royal National Health Service Foundation Trust, Faculty of Biology, Medicine and Health, The University of Manchester, Manchester, United Kingdom; ^3^Geoffrey Jefferson Brain Research Centre, The Manchester Academic Health Science Centre, Northern Care Alliance National Health Service Group, University of Manchester, Manchester, United Kingdom; ^4^Lydia Becker Institute of Immunology and Inflammation, The University of Manchester, Manchester, United Kingdom

**Keywords:** concussion, neuroimmunology, microglia, neurodegenenerative diseases, inflammation, mild TBI

## Abstract

Mild traumatic brain injury (mild TBI), often referred to as concussion, is the most common form of TBI and affects millions of people each year. A history of mild TBI increases the risk of developing emotional and neurocognitive disorders later in life that can impact on day to day living. These include anxiety and depression, as well as neurodegenerative conditions such as chronic traumatic encephalopathy (CTE) and Alzheimer's disease (AD). Actions of brain resident or peripherally recruited immune cells are proposed to be key regulators across these diseases and mood disorders. Here, we will assess the impact of mild TBI on brain and patient health, and evaluate the recent evidence for immune cell involvement in its pathogenesis.

## Mild Traumatic Brain Injury

Traumatic brain injury (TBI) is a term used to include a spectrum of insults resulting from mechanical injury to the brain. TBI includes injuries that range from severe, with open skull injuries and major parenchymal disruption, to the mildest form of TBI, often termed concussion. Although widely used in everyday language, the term concussion is now less commonly used in medical and scientific terminology, as it lacks diagnostic precision and does not refer to underlying pathological processes (1, 2). Therefore, mild TBI is the preferred term and will be used throughout this review (1, 2). It is estimated that TBI affects 69 million individuals each year world-wide, with the vast majority of cases being mild TBI (3, 4). The main causes of mild TBI are motor accidents, falls, assaults, active-duty of soldiers, and domestic violence (5, 6). Recently, greater attention has been given to this condition due to the high prevalence of mild TBI among young athletes in relation to their involvement in collision sports such as American Football, soccer, and rugby (7).

Mild TBI is a physiological disruption of brain function and occurs due to mechanical distortion of brain tissue, most commonly from a blow to the head, but can also be caused by a blast injury frequently seen in soldiers serving in a war zone (7, 8). Rapid rotational velocity/acceleration (inertial loading) is thought to be a key component of injury (9–11). The underlying pathophysiology of the injury remains poorly understood, as availability of human post-mortem brain tissue for examination from this typically non-lethal injury is limited (12). Although referred to as “mild,” individuals can still experience a variety of physical, emotional and cognitive problems, including sleep disturbance, increased anxiety, and depression (4, 7, 13, 14).

**Figure 1:**
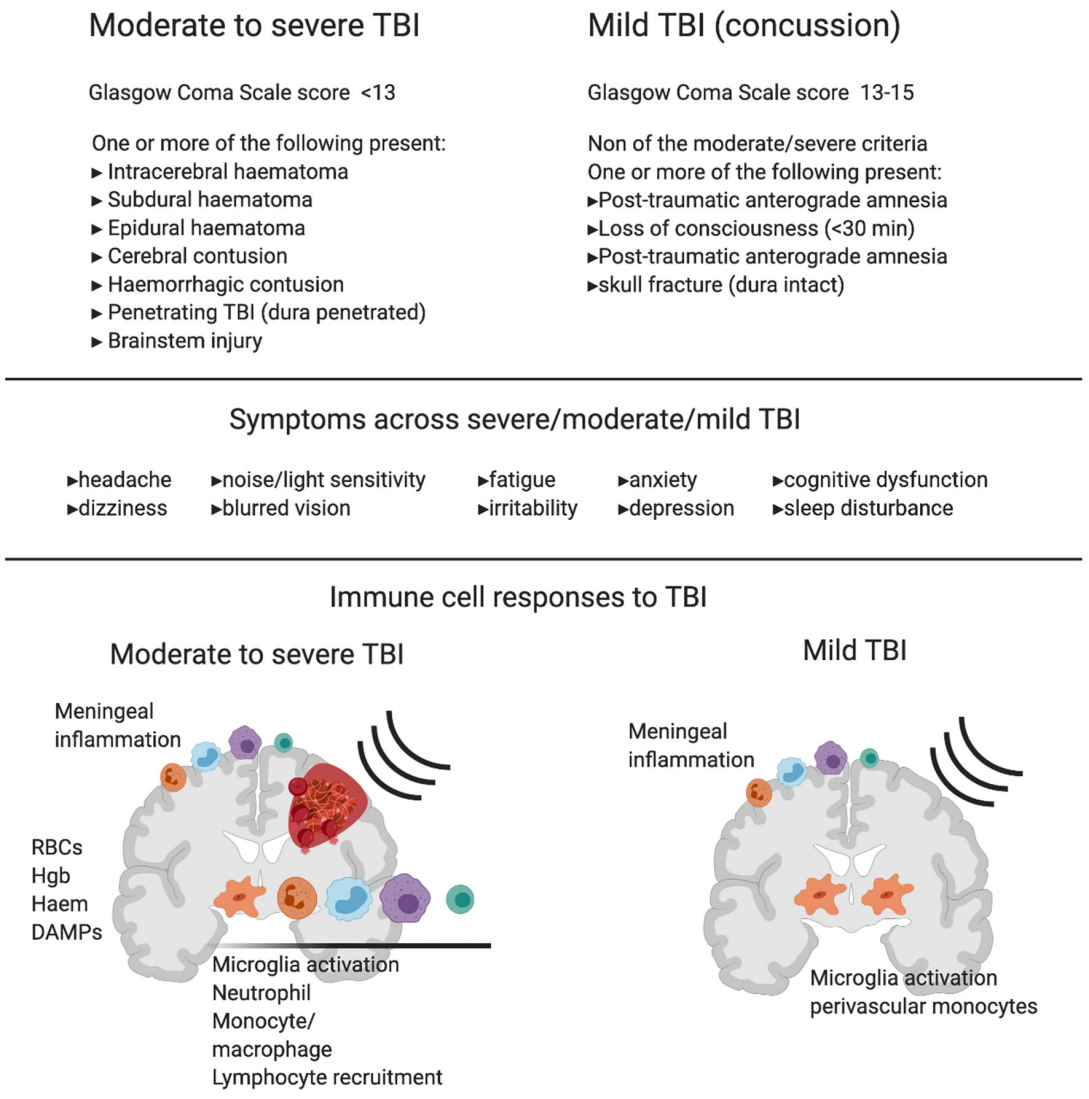
Diagnostic criteria, symptoms and immune cell involvement in moderate to severe traumatic Brain Injury (TBI) in comparison with mild TBI. Commonly used diagnostic criteria in moderate to severe TBI compared to mild TBI shows the major clinical difference between the two reflects hemorrhage or clear contusion in the brain. Symptoms are shared across mild, moderate and severe TBI with increasing likelihood of symptom occurrence and severity with increasing injury. Schematics represent immune response in moderate to severe TBI (left) and mild TBI (right). In moderate to severe TBI in humans and animal models, there is clear evidence for resident microglia activation and recruitment of macrophages, dendritic cells, neutrophils, B cells and T cells, and meningeal inflammation. In addition to active recruitment mechanisms, peripheral immune cells can infiltrate with frank hemorrhage alongside red blood cells (RBCs) and the release of hemoglobin (Hgb), Haem, and other damage associated molecular patterns (DAMPs), which are one set of initiators of the immune response. In contrast, in mild TBI there is little evidence of infiltrating immune cells to the brain tissue in humans or animal models that do not produce hemorrhage or skull opening. In mild TBI, there is evidence of meningeal inflammation, microglial activation, and some monocyte/macrophage recruitment to the cerebrovasculature.

Diagnosing mild TBI and its severity is usually based on the loss of consciousness duration (<30 min), the Glasgow coma scale score (13–15), post-traumatic amnesia duration (momentarily to <24 h), and a lack of intracerebral/subdural/epidural hematoma, cerebral, or hemorrhagic contusion, penetrating TBI (dura penetrated), subarachnoid hemorrhage or brainstem injury (2) (Figure 1). Mild TBI is still a heterogeneous insult, and there can be major variation in the likelihood of significant neuropathology and varying symptoms including blurred vision, confusion, dizziness, focal neurological symptoms, headache, and nausea (2, 7, 8). Whilst most patients recover and return to their normal self, the clinical outcome of concussion is hard to predict. This is because of the heterogeneity of initial trauma, the inability to quantify disease severity and the likely initiation of complex pathogenic pathways (15). Even though men are at greater risk of mild TBI due to greater participation in high-risk activities, studies have shown that females are at greater risk of poor outcomes (16–19) and further research of both sexes is needed to characterize the nature of sex-dependent injury and recovery (20). In addition, pre-existing health conditions, age, genetic background (21), and alcohol or substance abuse also influences recovery and leads to differences in clinical outcome between patients (22).

The following review will focus solely on consequences of mild TBI in adults, that would be commonly be referred to as concussion. There is a vast and important literature on more severe TBI that includes evidence of hemorrhage and parenchymal injury and these injuries are defined as moderate or severe TBI. Clinically, moderate or severe TBI is diagnosed by neuroimaging and preclinical modeling of these injuries is much more common than mild TBI, due to the production of frank and measurable tissue damage. As a result, there is a large literature on neuroinflammatory and immune mechanisms that drive both injury and repair in these insults (23–25). Much less is known about the pathology of mild TBI with no overt contusion or hemorrhage in the pathology, and here, a different immune response is likely to occur. The current review will explicitly refer to evidence of the immune response after human mild TBI, in animal models with high translational relevance to mild TBI (without compromising the skull and no evidence of hemorrhage) and studies that investigate patients with a history of head injury through sport.

## Evidence for Mild TBI as a Risk Factor for Long-Term Problems

Mild TBI is now recognized as a major public health concern as clinicians and researchers are becoming more aware of the dangers and potential long-term consequences associated with this type of head injury (26). In most mild TBI cases, acutely reported symptoms resolve within 3 months; however, a small proportion of patients continue to suffer life disrupting symptoms (27–29). A range of factors, not necessarily directly reflecting injury severity, are associated with poor outcome following mild TBI, including previous neurological or psychiatric problems and whether the patient had suffered a previous head injury (27, 28). Indeed, patients with a history of mild TBI can experience changes in emotions or behavior, often expressed by increased anxiety and depressive like behaviors (2, 4, 7, 13, 14). Most studies investigating multiple head injuries over a sustained period are derived from participants of contact sports. These patients may differ greatly from those who suffer a mild TBI as a one-off event, not only in the nature of the head injury but also their lifestyle and pre-morbid traits (30). Currently, there is major interest in mild TBI/concussion due to its prevalence in sports such as the National Football League (NFL), rugby, and soccer, where the risk of head injury is high. Single concussive events in these sports can result in the same myriad of symptoms as a one-off mild TBI in the general population, and may trigger that same initial pathological response; however, it is the accumulation of injuries and their long-term effect on mood and neurodegenerative outcomes that is often assessed in these athletes.

### Effects of Repeated Mild TBI in Contact Sports

In contact sports, diagnosed mild TBIs/concussions and even head impacts that are frequent but do not cause noticeable immediate injury, such as heading a soccer ball, are now being investigated as risk factors for poor long-term brain health (31). In a population of retired rugby players, the prevalence of major depressive disorder was significantly higher compared to other retired sportsmen (7). Another study investigated professional NFL players and found a link between recurrent concussion and diagnosis of lifetime depression and suggested that the prevalence of depression increases with the number of past mild TBIs (32). Indeed, retired players that reported either one to two, or three or more previous concussions were 1.5 and three times more likely to be diagnosed with depression, respectively, compared to retired players with no history of mild TBIs (32). Regardless of the type of contact sport, diagnostic test scores for major depressive disorder increases with the number of mild TBIs (7).

In addition to emotional disturbances, mTBI is associated with a risk of developing a number of neurodegenerative conditions (33). A series of studies retrospectively investigating a cohort study of former professional footballers [Football's Influence on Lifelong health and Dementia risk (FIELD)] investigated the link between at dementia pathology, mortality and mental health and suicide in ex footballers (soccer players) (34). Mortality from neurodegenerative disease was higher and mortality from other common diseases lower among retired professional soccer players than among matched controls (35). Surprisingly, in these cohorts, hospital admissions for common mental health disorders were lower than population controls, with no difference in suicide, despite evidence of neurodegeneration (36). Outside of professional athletes, a population-based administrative health cohort study, in more than 47,000 cases of mild TBI showed mild TBI was associated with an increased risk of diagnosis of attention-deficit hyperactivity disorder, mood and anxiety disorders, dementia and Parkinson's disease later in life (37).

The majority of evidence suggests that mild TBI can be detrimental to mental health, but also carries increased risk of developing epilepsy and neurodegenerative disorders, such as Alzheimer's disease, Parkinson's disease, and chronic traumatic encephalopathy (CTE) (2). Significant neurodegeneration observed in retired athletes has been linked to repeated mild TBI at a younger age and mortality from neurodegenerative disease is significantly higher among former professional soccer players than in matched population controls (35). Within this group, Alzheimer's disease as the primary or a contributory cause of death was responsible for the largest increase of deaths (35). In a separate study, there was increased risk and early onset of ALS in professional players from Italian soccer teams (38).

Post-mortem studies on former contact sports athletes show a high prevalence of CTE; a progressive neurodegeneration associated with repetitive head trauma (39, 40). Interestingly, neuropathological severity of CTE seemed to increase in accordance with the level of play and almost all cases had behavioral or mood symptoms or both, cognitive symptoms or signs of dementia (39). Post-mortem brains from U.S. military veterans that have been exposed to blast exposure and/or concussive injury display CTE neuropathology that is similar to the pathology observed in former athletes (41).

These studies add to the growing evidence to suggest that a history of mild TBI is a risk factor for the development of pathological neurodegeneration. They have also brought much needed attention to the dangers of head injury, in general. Research is now focused on understanding the underlying pathology of both single and accumulated mild TBIs, and what can be learned from each individual instance to prevent long-term problems.

### Understanding Distinct or Overlapping Mechanisms of a Single, Repeated, or Life-Long History of Mild TBI

The *Lancet* Commission on dementia prevention recently added TBI as potentially modifiable risk factor for dementia (26). In this important document, a combination of studies relating to severe TBI, those investigating concussion, or a career in professional contact sport, are cited as why *traumatic brain injury* is considered a risk factor for dementia and neurodegenerative disease (26, 33). As detailed above, severe TBI can be quite different to mild TBI. However, the role of neuroinflammation, propagated by the immune system, is a likely modifiable regulator in both. Even when focusing on mild TBI research alone, there are still many challenges to assessing the role of the immune system. Complicating factors include: heterogeneous pathology and symptoms (2), diffuse injury across brain regions (10, 42) and the overlapping research conclusions between one-off mild TBI and multiple injuries sustained by professional sport participation. However, the investigation of the immune response to discrete injuries will undoubtedly lead to increased understanding of the dangerous cumulative effects of multiple mild TBIs. Indeed, much of our understanding of the mechanisms of mild TBI, and the role of the immune system, is derived from animal models of injury. A combination of clinical and preclinical studies in the acute setting can provide insight into both mild TBI in the general population and those accumulated in professional sport.

The following sections will outline what is known about the immune system's response to CNS injury in general, and why aspects of the immune system have become considered drivers of neurodegenerative disease, of which head injury is now considered a risk factor. This will provide context for the review of the current literature for immune involvement in the pathology of mild TBI.

## Positioning of the Immune System Within the CNS

Immune cells are present throughout the adult CNS (43). Microglia are a type of tissue resident macrophage and are the major immune cell type (44). Microglial cell bodies and their processes cover every cubic micrometer of the brain during constant surveillance activities (45, 46). Recent work shows that, of the total number of immune cells in the brain, ~80% are microglia, with the remainder comprised of barrier-associated macrophages and cell types more traditionally associated with the periphery such as neutrophils and T cells (44, 47–49). Microglia are highly plastic and defend the brain against external challenges. Pattern recognition receptors (PRRs) are scattered along their membrane, by which they can recognize pathogen-associated molecular patterns (PAMPs) and host-derived danger-associated molecular patterns (DAMPs), making microglia equipped with the tools to evoke a rapid, fine-tuned inflammatory response to immunological challenges (50). Microglia phenotype and morphology are determined by their local environment and several concepts relating to their function, such as “homeostatic,” “primed,” “trained,” or “tolerant” microglia, have emerged from experimental models (51). Microglial priming is defined as a prolonged and exaggerated immune response resulting from an acute inflammatory event in an ongoing inflammatory environment (52). Innate immune memory is associated to cell reprogramming following a primary immune stimulus that leads to increased (trained) or decreased (tolerant) responses to a secondary inflammatory stimulus (53). These concepts are important in the context of mild TBI as repeated head injuries lead to greater risk of poor outcome (32, 54). Although not defined as immune cells *per se*, astrocytes, oligodendrocytes, and endothelial cells all perform various functions that are critical to the immune response (55–58), and the important actions specific to these cells in mild TBI are reviewed elsewhere (59–62).

Neuroinflammatory cascades rely on the activation of the inflammasome, a protein complex, consisting of caspase-1, apoptosis-associated speck-like protein (ASC) and nod-like receptor protein (NLRP1 or NLRP3) (63, 64). Common microglial pathways activated upon the detection of a challenge involve NF-κB, which is a pro-inflammatory transcription factor that stimulates cytokine release in conjunction with the inflammasome (50). Metabolic changes within microglia also sustain or restrain inflammation (65). Rapid motility, reactive oxygen species (ROS) and cytokine production require quick energy utilization through glycolysis and fatty acid synthesis (65–67). In contrast, anti-inflammatory microglia require efficient energy production utilizing oxidative phosphorylation for transcription of ATP-dependent tissue repair genes, reduce ROS, perform amino acid and fatty acid oxidation to produce growth factors, including polyamines and prolines, and to support mitochondrial respiration (65–67).

In the event of CNS injury, microglia reduce their ramifications and extend cell processes to the site of injury, helping to maintain the integrity of critical CNS barrier structures such as glial limitans and vasculature (68–70). Moreover, they increase their migration to damaged brain sites and become phagocytic to clear cell debris (71–73). Microglia and other resident immune cells can be joined by their infiltrating counterparts from the circulation, such as neutrophils, monocytes, and lymphocytes, depending on the severity of injury (43, 71, 73–79). These peripheral cells are recruited through a multitude of mechanisms, including endothelial and microglial signaling, and can enter the brain through the compromised blood brain barrier, circumventricular organs or other brain blood interfaces, such as the meninges (80, 81).

In the context of human mild TBI, it is unknown whether circulating immune cells are recruited to the brain in patients, and closed head animal models provide differing results depending on induction and severity of the injury [see below]. Recruitment of immune cells may vastly affect progression of pathology, as the actions of these cells can differ compared to their resident counterparts (43). Furthermore, evidence suggests that infiltrating immune cells influence resident microglia populations, which may have long lasting consequences for injury outcomes (74, 82–85).

Here, it is again important to distinguish between TBI with parenchymal hemorrhage (and associated animal models) and mild TBI, as hemorrhage is likely to create a type injury and immune response that is completely distinct to that of injuries with no bleeding (Figure 1). For example, extravasated red blood cells (RBCs) are a source of multiple immune response triggers and DAMPs (86). RBCs are phagocytosed by immune cells, which drives an inflammatory phenotype in those cells (87), and a portion of which stay within the tissue, die and degranulate, releasing endothelins and oxygen free radicals (88). Extravasated RBCs also releases toxic Hgb which is oxidized to haem and acts as a DAMP to exacerbate the inflammatory response (89). A further metabolite, iron is also implicated in brain injury after hemorrhage (88, 90, 91). Thus, several stages of RBC lysis contribute to a type of brain injury not seen in mild TBI without parenchymal bleeding. For information on the immune response to TBI including hemorrhage, we would like to point readers to the following excellent reviews on the topic (23–25).

In mild TBI, whether immune cells from the periphery are recruited or not (see below), microglia and the other resident immune cells are present and can respond rapidly to changes in the brain (44, 47–49). In the wider field of neuroimmunology, the interest in the microglia-mediated immune response during brain injury and disease has risen exponentially in the past decade, mainly due to genome wide association studies that implicate many microglial genes as risk factors for neurodegenerative disease (92, 93). It is here, in the brain's immune response, where mild TBI and the risk of cognitive decline and neurodegeneration may meet.

### The Immune System's Role as a Driver of Neurodegeneration

To understand if the immune response to mild TBI increases the risk of neurodegenerative disease, it is important to understand the known role of the immune system in neurodegeneration. The CNS and innate immune system continuously modulate each other through a sophisticated bidirectional crosstalk (43, 94). Under pathological conditions, disrupted communication may result in an inflammatory response. When the inflammatory response of CNS resident immune cells remains unresolved, this may lead to initiation, propagation, and progression of tissue damage, ultimately resulting in neurodegeneration (50). The immune system may therefore be a driver of neurodegeneration, in general (95–98) and mild TBI's activation of the immune system may be a causative trigger, although this is yet to be formally demonstrated.

Many neurodegenerative disorders display concurrent and chronic alterations in immune function and signaling. However, there is now strong evidence that immune dysregulation can be a direct cause of neurodegenerative disease. Somatic mutation specifically in the erythro-myeloid progenitor lineage from which microglia derive can drive late-onset neurodegeneration in mice (56). More recently, biallelic mutations in *NRROS* (Negative Regulator Of Reactive Oxygen Species), which is necessary for TGFB-1 signaling in microglia, were found to cause an early onset lethal microgliopathy in humans (99, 100) and NRROS-deficient (Nrros-/-) mice show neurodegeneration (101), defects in motor functions and die before 6 months of age (102). Together, these data show that microglia-specific alterations can cause neurodegenerative disease, confirming that the well-documented immune response to neurodegeneration may not solely be secondary to injury.

Several genes involved in the immune system, and particularly microglia, have been identified as risk factors for the most common form of neurodegenerative disease, Alzheimer's (103–105). The APOE gene, encoding apolipoprotein (Apo)E (106) is mainly expressed in the brain by microglia and astrocytes and is component of Aβ plaques and promotes Aβ aggregation and deposition (107). The three major human isoforms, apoE2, apoE3, and apoE4, are encoded by different alleles and differ in their effects on AD risk and pathology, with one APOE-ε4 allele increasing AD risk 3-fold and two APOE-ε4 alleles increasing AD risk by 12-fold (108). In addition, APOE-ε4 is also implicated in Dementia with Lewy bodies and Frontotemporal dementia (97). Another molecule implicated in AD is the triggering receptor expressed on myeloid cells 2 (Trem2) and is highly expressed in microglia in the brain and important for microglial, phagocytosis, proliferation and environment sensing (109). Single-nucleotide polymorphism (SNP) mutations in Trem2 drastically increase the AD risk (110), estimated to be with a 3.0- to 4.5-fold (96). Moreover, homozygous Trem2 variants have been proposed to be causal for Frontotemporal Dementia (FTD) or linked to increased FTD risk (111). A recent genome-wide meta-analysis identified new loci and functional pathways influencing Alzheimer's disease risk that localized to immune-related tissues and cell types (microglia), highlighting the role of the immune system and its principle brain-resident cell in neurodegenerative disease (92). In sum, the immune system and its dysfunction are now strongly implicated in neurodegenerative disease, for which mild TBI (particularly repeated mild TBI) is proposed as a risk factor. We will now summarize how mild TBI may activate the brain's immune system, potentially linking it to long-term neurodegeneration.

## The Immune Response to Mild TBI

To reiterate the focus of this article, we will review the known immune response to injuries that are defined as mild TBI or concussion and their relevant animal models. This would therefore exclude brain injuries with intracerebral, subdural, epidural hematoma, cerebral or hemorrhagic contusion, penetrating TBI (dura penetrated), subarachnoid hemorrhage or brainstem injury (2) (Figure 1). This definition describes a population of patients that have potential pathology too subtle for standard imaging, but represents up to >80% of those that suffer a TBI (3). Animal models representing this “mild” situation are less common as researchers investigating brain injury are in search robust, reliable, modifiable readouts to demonstrate mechanisms and new treatment options; such animal models often employ craniotomy and/or hemorrhage and contusion and would be considered moderate to severe TBI in the clinic. If the immune response to mild TBI is a factor in pathology, it is important to understand how this may be initiated in a brain without bleeding or overt lesion.

### Triggers of the Immune Response in Mild TBI

**Figure 2:**
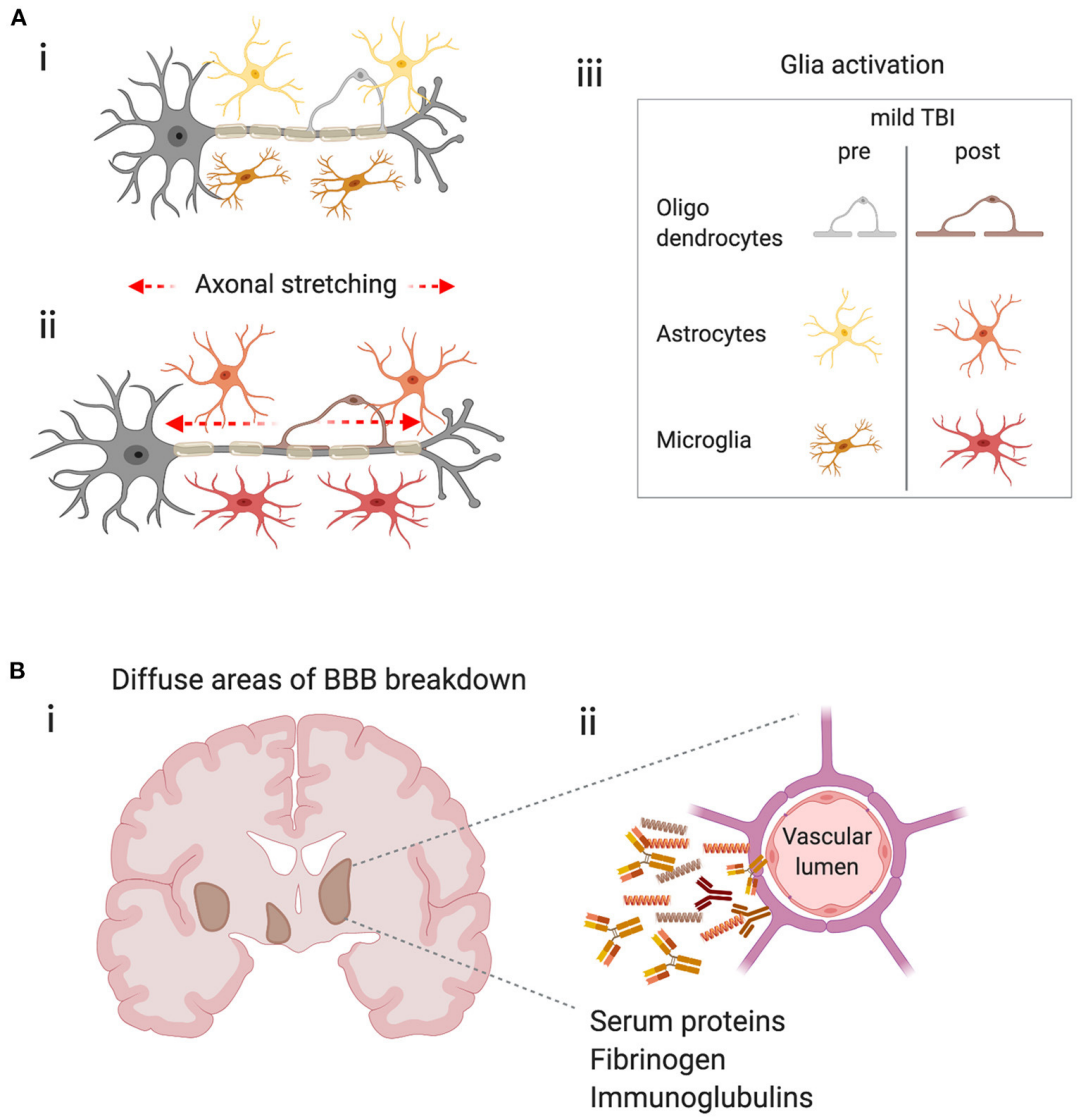
Potential triggers of the immune response in mild TBI. **(A)** Physical forces lead to stretching of axons, axonal injury and glial activation. (i) Schematic representing a neuron with its myelinated axon surrounded by glia in the healthy brain. (ii) Represents the stretching of axons due to physical forces during mild TBI and the subsequent glial activation (iii) Key–indicates glial subtypes hypothesized to be activated after axonal stretching. **(B)** Diffuse blood-brain barrier (BBB) in the brain after concussion leads to extravasation of harmful molecules to the parenchyma. (i) Schematic shows a coronal section of a human brain with representations of diffuse BBB breakdown after mild TBI (brown areas). (ii) Enlarged image shows compromised cerebral blood vessel leaking molecules that may trigger inflammation and glial activation.

The immune system is ubiquitous in the CNS and is equipped to respond to brain tissue injury after mild TBI. The injury produced after a closed-head impact is the result of physical movements of the brain within the skull, including acceleration and differential inertial loading (10, 11). Physical forces are thought to lead to axonal injury due to excessive regional stretching of axons (112) (Figure 2A). Using head kinematics from athletic events, modeling showed that the brain can be described as a hyperviscoelastic medium and deformation is most sensitive to specific frequency oscillations, particularly in deep brain regions, and is aligned with areas of pathology after mild TBI (10).

Stretching of axons is the leading hypothesis as to why white matter appears most sensitive to mild brain injury. However, from the view of the immune system, microglial cells are also sensitive to mechanical signals (113, 114). Viscoelastic testing of individual CNS cells showed that other glial cells, such as astrocytes and Müller glia are twice as soft as neurons and act as compliant structures surrounding the neuronal cells, and are described as “cushioning material” (115). This opens the possibility that glia, in general, are differentially susceptible to forces produced in the brain during mild TBI. Mechanical changes in glial cells may physically protect neurons from initial mechanical damage, but then subsequently become activated and produce factors that drive neuronal pathology (Figure 2A). Alternatively, glia may be bystanders, solely responding to neuronal injury when the force is big enough to affect neurons directly. In either scenario, an aberrant microglial response due to either genetic factors, such as APOE genotype or TREM2 mutations (see above), or environmental influences, such as inflammation due to infection, poor diet or obesity may contribute to concussive injury as a risk factor for neurodegenerative disease (26).

Physical forces may also result in damage to the blood-brain barrier (BBB), a specialized endothelial barrier that tightly regulates molecular and cellular movement into the brain (116). BBB breakdown is long associated with moderate to severe brain injury (Figure 2B) but has recently been shown to be present in clinically relevant models of mild TBI. In awake mice, 24 h after a closed-head impact injury, serum albumin extravasation and evidence of myeloid inflammatory cell infiltration due to BBB breakdown was localized to the lateral surface of the ipsilateral perirhinal cortex adjacent to the impact contact zone (117). In a swine model of head rotational acceleration and in the absence of hemorrhage or other focal pathology, disruption of the BBB was found 6–72 h after injury, by extravasation of serum proteins, fibrinogen and immunoglobulin-G (12), confirming earlier mouse studies (118). BBB disruption was consistent with the biomechanical insult as extravasated serum proteins were observed at interfaces between regions of tissue with differing material properties, including gray–white matter boundaries and periventricular and subpial regions which overlapped with regions of axonal pathology in the white matter (12). This highly relevant model of mild TBI provides insights to subtle, yet significant pathology that is likely to trigger immune responses, yet be undetectable in humans through standard clinical imaging techniques.

Fibrinogen is a central blood coagulation protein that is deposited in the CNS after BBB disruption (62, 119) and is found in brain tissue in models of mild TBI described above (12). Fibrinogen induces encephalitogenic adaptive immune responses and peripheral macrophage recruitment into the CNS leading to demyelination in models of multiple sclerosis (120). Interestingly, fibrinogen deposition in the CNS affects many processes across diseases, including suppression of remyelination through OPC function (121) and induction of microglia-mediated spine elimination, leading to cognitive deficits in a model of AD (122). Both white matter damage and cognitive deficits are described in mild TBI. Currently, a promising advance for treating pathologies in involving fibrinogen deposition, is the generation of a monoclonal antibody 5B8, that selectively inhibits fibrin-induced inflammation and oxidative stress without interfering with clotting and shows efficacy in animal models of MS and AD (123). Whether such strategies will be employed to improve pathology and cognitive and emotional deficits after mild TBI remains to be seen.

More data are required to understand the initial triggers of the immune response in mild TBI, and a combination of neuronal damage through mechanical stretching, direct mechanical damage of glia and endothelial cells and BBB-breakdown are all likely to play a role. Irrespective of the initial driver of brain tissue damage, patient studies indicate there is an active immune response in those individuals exposed to mild TBI injury.

### Clinical Evidence of the Immune Response to Mild TBI–Serum Biomarkers

An important area of brain injury and disease research is the search for blood biomarkers that reflect brain processes, to predict disease course and used as readouts to assess utility of interventions to improve outcome for patients. Recently, a range of proteins, including neurofilament light (NfL) polypeptide released from damaged axons, have been proposed as viable biomarkers of mild TBI (60, 124, 125). The brain's immune response to mild TBI is also evident in the blood, as elevated c-reactive protein (CRP) levels at admission are independently associated with the increased risk of persistent psychological problems and cognitive impairment (29). Plasma interleukin (IL)-2 and IL-6 levels are also significantly higher for mild TBI patients compared with orthopedic injury controls, indicating a brain-specific injury initiated an immune response that is present in the periphery (126). Elevated IL-2, 24 h after injury, is associated more severe early post-concussive symptoms, while elevated plasma IL-10 level at 6 months is associated with more severe posttraumatic stress disorder (PTSD) and mood scores (126). Overall plasma levels of IL-1ß, IL-4, IL-6, and IFN-γ are reduced at 6 months compared to acute levels, indicating a subsiding of inflammation caused by the initial injury. Interestingly, plasma levels of the anti-inflammatory cytokine IL-10 have also been shown to be predictive of a mild TBI vs. a moderate-to-severe TBI (as defined by hemorrhage on CT) (127), again highlighting the different immune responses between injury types. Further to immune mediators detected in the plasma, complement pathway proteins (key mediators of the immune response) are elevated in astrocyte-derived exosomes in the plasma within seven days of a mild TBI (128). In the non-acute setting, a study of veterans with a remote history of mild TBIs found an association between concentrations of TNF-α and post-concussive syndrome (PCS) and PTSD symptoms (125). The total number of mild TBIs correlated with exosomal and plasma NfL levels and plasma IL-6 (125). These results indicate a persistent elevated neuronal and neuroinflammatory response many years after mild TBI.

Serum biomarkers of brain injury and the immune response are now being collected in a range of settings, from single injuries to chronic sport related mild TBI in male and female participants (129). Although, the peripheral immune response to more severe TBI is well documented (24, 64, 130), the data specifically in mild TBI highlights that circulating immune mediators are also present and may be involved in symptom progression and resolution.

### Clinical Evidence of the Immune Response to Mild TBI–Neuroimaging

The immune response can also be measured indirectly in the brain by clinical neuroimaging. The meninges, a protective layer of membranes surrounding the brain, have gained much attention in the field of neuroimmunology as they are the home of the brain's lymphatic system (131, 132) and a variety of immune cell populations (44, 47, 49) which, in animal models, can regulate brain function in health (133), disease (134, 135), infection (136), and recovery from TBI (137). In patients with mild TBI, enhancement of the meninges on post-contrast images obtained by fluid attenuated inversion recovery (FLAIR) magnetic resonance imaging (MRI), show abnormalities that may reflect inflammation in the immune cell rich meningeal membranes (138, 139). Meningeal immune cell-mediated inflammation can influence neuronal populations in the brain parenchyma in mice, resulting behavioral changes that are also associated with symptoms of mild TBI, such as anxiety (133, 137). As yet, it is unknown whether meningeal inflammation in human mild TBI plays a role in emotional symptoms seen after injury.

In the context of athletes with a history of mild TBI, initial neuroimaging studies of former retired NFL players reported cognitive deficits that were correlated with white matter abnormalities and changes in regional cerebral blood flow (140) and that mild TBI is a risk factor for development of mild cognitive impairment (140). Following these findings, work began to show that neuroinflammation in the brain tissue proper was also a key component in those at risk from concussive-symptoms.

It is well-known that brain injury and disease cause a change in the functional state of microglia, the major cell type in the brain responsible for neuroinflammation (141). Neuroinflammation is associated with the *de novo* expression of the mitochondrial 18 kDa translocator protein (TSPO), a binding site for which many selective high-affinity compounds for PET imaging have been developed (142). During brain injury or disease, TSPO is mainly expressed in microglia but can also be detected in astrocytes (51, 143, 144). Increased regional TSPO expression in the brain typically covaries with disease state and activity and is proposed to be a non-diagnostic biomarker and secondary to disease etiology. As result, many clinical studies use *in vivo* measurements of TSPO expression as a biomarker of disease progression or therapeutic efficacy (142).

In former NFL players, early studies showed significant increase in binding of the radio-ligand [^11^C]DPA-713 to TSPO in several brain regions, such as the supramarginal gyrus and right amygdala, compared to age-matched, healthy controls, indicating neuroinflammation in those areas (145). The same former players had varied performance on a test of verbal learning and memory (145). Interestingly, studies performed in much younger, active and recently retired NFL players with a self-reported history of mild TBI also revealed increased [^11^C]DPA-713 binding to TSPO in eight of the 12 brain regions examined, but did not differ from control participants in regional brain volumes or in neuropsychological performance (146). This opened the possibility of using TSPO binding a biomarker for brain changes in the younger concussed brain, prior to cognitive decline (146). Postmortem studies of former NFL player confirmed that repeated head injury is associated with chronic activation of microglia (147). In addition, the duration of repeated head injury exposure, as defined by the years of football played, predicted greater density of CD68 positive inflammatory microglia in NFL players with and without CTE pathology and that increased neuroinflammation was related to the risk of a subject being diagnosed with dementia, independent of age (147). Together, these studies show that the immune response is detectable in patients before cognitive changes, and persists throughout the lifetime of those exposed to concussive injury.

Other strategies to assess immune cell activation in response to concussion injury include the quantification of protein markers in the CSF, such as soluble TREM2 (sTREM2). TREM2 is a transmembrane receptor of the immunoglobulin superfamily highly expressed in microglia which multiple ligands (148). TREM2 surface expression rapidly declines on activation of microglia which is partly due to sequential cleavage of membrane-bound TREM2 by proteases that release soluble TREM2 (sTREM2) (149). As result, sTREM2 has been hypothesized to be predictive of microglial activation if detected in the CSF. In former NFL players, sTREM2 levels were higher in their CSF compared to controls and were associated with higher t-tau concentrations, which are thought to be a major factor in neurodegeneration (150). However, in patients with TREM2 mutations with AD and FTD, sTREM is reduced in the CSF (151). Other studies have shown increases of CSF TREM2 in AD dementia (152, 153), or increase in MCI-AD but not AD dementia (154). Therefore, the use of sTREM as a direct biomarker for microglia activation is yet unproven, and the biological role sTREM2 in neurodegeneration is unknown.

Patient studies investigating the microglial response to mild TBI have largely focused on individuals with a known history of head injury, such as professional athletes. Recently, TSPO expression was assessed after a single-event mild TBI in patients without signs of structural damage 1–2 weeks and at 3–4 months after injury (155). Importantly, patients were not included if they showed any intracranial lesion on the initial computed tomography scan or had a history of head trauma with loss of consciousness. Using the single photon emission computed tomography tracer 123I-CLINDE, persistent TSPO upregulation was found at 3–4 months post-injury, even in patients with good clinical recovery (155). This is consistent with the data from active or recently retired NFL players that had increased TSPO binding, but no change in neuropsychological testing (146). Clearly, it is unknown whether a single head injury and subsequent TSPO increases is comparable to a history of head injury and persistent TSPO increases, and whether they have the same long-term consequences, but it is evidence that a sub-chronic immune response occurs in even mild injury with resolved short-term clinical symptoms.

### The Immune Response in Animal Models of Mild TBI

Models of traumatic brain injury have provided insight into multiple mechanisms of tissue damage. Even for “mild” TBI, animal models vary widely, from impacts direct to the brain's surface in fixed-head, anesthetized animals to injury with no surgery or anesthesia with forces that allow head rotation (59, 156). Each model (even similar in description), in different laboratory's hands can lead to different etiologies and severities, which should be considered when assessing the literature. Often the least “clinically relevant” model can lead to the most useful mechanistic insight, and those most likely to best recapitulate human concussion should inherently be the most heterogeneous in their outcome. Both extremes are now being used to explore the role of the immune system in mild brain injury. We will not attempt to review the different models of injury here, but we point the reader to excellent reviews in the field (59, 156). As mentioned previously, we will focus our attention on closed head injury models that most closely resemble clinical mild TBI, at least in as much as do not use any type of craniotomy or produce frank tissue damage or hemorrhage. We will assess the only those models that are “closed skull” with no observable bleeding and investigate the immune reaction to injury. These models are relatively few compared to the wider TBI literature but may offer key insights to pathology.

In a pig model of closed-head rotational acceleration, biomechanical loading parameters can be replicated that are thought to underlie neuropathology of mild TBI in humans (157). Pigs have a large gyrencephalic brain and a gray to white matter ratio similar to humans, which is important as diffuse axonal injury in white matter is believed to be the principal pathology of closed-head diffuse brain injury (42, 158). Using these models, changes in microglia morphology around compromised neurons was described as early as 15 min after injury, potentially allowing microglia to influence the evolution of subsequent neuronal damage (159). Evidence from many other models of CNS injury suggest that initial actions of microglia are protective (69, 160–162), though this is unknown in the context of mild TBI. In the same pig model, investigation of later time points after injury, found subtle neuronal changes in the hippocampus (163, 164) and microglia had increased signs of activation up to 1 year after injury (164). Just as acute microglial actions at sites of CNS injury are proposed to be beneficial, long-term microglial-mediated inflammation is thought to be detrimental and may be involved in the long-term complications associated with mild TBI (165–173). Longer-term activation of microglia was also seen in non-surgical, diffuse closed-head injury in mice, in a model characterized by an impact as well as linear and rotational acceleration (174). Thirty days after injury, multifocal, bilateral axonal damage with neuronal death in the hippocampus was detected, microgliosis was prominent and neurobehavioral deficits observed in spatial learning/memory and socialization (174). Indeed, a range of studies in appropriate rodent models show microglial activation across multiple time points, particularly in white matter tracts (165–172) and may be a result of rotational stress or reduced cerebral blood flow (175, 176).

To investigate the causality of inflammation and the immune response on mild TBI deficits, multiple studies have manipulated these pathways and investigated behavioral outcome measures. For example; selective, small molecule inhibition of acute pro-inflammatory cytokines and chemokines, nanopeptides targeting apoE mediated the neuroinflammation and minocycline administration all reduce microglial activation and improve neurological outcome after mild TBI, respectively (177–179). Hippocampal microglia activation is attenuated by inhaled nitrous oxide and correlates with improved performance on memory tasks (180) and statins reduce pro-inflammatory cytokine gene expression in the brain, reduce microglial activation and improve functional neurological deficits after mild TBI (181). Nilvadipine, a tyrosine kinase inhibitor, suppresses inflammation and restores spatial memory deficits (182) and administration of Carnosic acid, and inhibitor of NF-kB, significantly improves motor and cognitive function and reduces microglia activation in white matter tracks in a mouse models of repetitive mild TBI (183). The neuronal expression of pro-inflammatory mediator complement receptor C5a is upregulated after mild TBI and is dependent on TNF (184). Mice lacking a functional alternative pathway of complement activation have reduced neuronal cell death after mild TBI (185) and removal of the protective neuronal-derived complement protein CD59, worsens neurological outcome seven days after mild TBI in mice (186).

These studies show that, like in humans, inflammation and microglial activation are prolonged after injury. Animal models show that the neuroimmune response correlates with cognitive deficits and may be modified to improve outcome, though more evidence of causal contribution of the immune system to behavioral deficits is needed.

Models of mild TBI are continually being refined. Recently, it was shown that a commonly used inhaled anesthetic, isoflurane, inhibits microglial ramification and surveillance *in vivo* (187), therefore potentially blocking the immediate immune response of the brain to injury when animals and anesthetized. The issue of anesthesia is prominent in almost all models of brain injury, but some have begun to produce concussive injuries in awake, restrained animals. In awake rats, using an injury paradigm with a bespoke helmet, injury produced memory deficits, and microglia activation after impact verses sham (188). Although, these studies remove the issue of anesthetic effects on the immune system, the stress induced by restraint of animals must be considered. Restrain habituation for the above study was performed for 3 days before injury, and stress induced inflammation may still be a contributing factor.

The onset and duration of inflammation driven by immune mechanisms after concussion is likely to play a key role in pathology. Activation of Toll-like receptor 4 (TLR4), which plays a fundamental role in pathogen recognition and activation of innate immunity, following repeated mild TBI is either beneficial or detrimental depending on the timing of activation. Administration of low dose LPS 1 day after injury was associated with a reduction in neuronal injury, a restoration of levels of myelin basic protein (MBP) and PSD-95 and no behavioral changes in locomotion, anxiety, depressive-like behavior or cognition at 3 months post-injury (189). Conversely, when LPS was given at 5 days after injury, it was associated with an acute increase in pro-inflammatory cytokine production, an exacerbation of neuronal damage and increased levels of aggregated and phosphorylated tau which led to a slight exacerbation of cognitive deficits and depressive-like behavior at 3 months (189). Due to the interest in the immune response after mild TBI, a natural avenue for therapeutics are anti-inflammatory treatments. However, modulating the immune response may require a strict, and yet unknown, time course. The strength of immune modulation should also be considered. In a model of AD, it was found that targeting the TLR4 receptor before onset of pathology by inducing either immune tolerance (4xLPS injections) or immune training/priming (1xLPS injection), either alleviates later B-amyloidosis with the former, or makes it worse with the latter (190). Such mechanisms must be considered when approaching inflammation after mild TBI, especially considering mild head injury's convergence with neurodegenerative disease (89).

In a model of lateral closed-head impact injury that uses momentum transfer to induce traumatic head acceleration in unanaesthetised mice, an abrupt onset and rapid resolution of a concussion-like syndrome is characterized by altered arousal, locomotor impairments and neurobehavioral deficits (117). The majority of brains in injured mice (~90%) had no evidence of frank hemorrhage or contusion but BBB breakdown was observed. An increase in the number of microglia and in infiltration of monocytes was observed 3 days after injury, though this was localized to the impact region (117). Uniquely, the study presented post-mortem brains from four teenage athletes in the acute-subacute period after mild closed-head impact injury and found, among other pathology, perivascular neuroinflammation in the form of haemosiderin-laden macrophages surrounding a small blood vessel (117). These data indicate that microglia may not be the only immune cell contributing to mild TBI pathology, as monocyte-derived macrophages from the circulation may enter brain tissue after mild injury, though more data is required to confirm this as an immune response to mild TBI pathology, in general.

Repeated mild TBIs cause more significant neurological damage than a single injury, including longer recovery time and a higher likelihood of subsequent brain injury (32, 54). It is hypothesized that priming of the immune response may play a role. Repeated mild brain injury produces greater microglial activation, anxiety-like behavior and impaired spatial memory compared to a single injury, in mice (191). In rats tested with projectiles and helmets, repeated injury produced a more significant and long lasting inflammatory response associated with microglial activation than a single injury (192). Delivering multiple mild impacts over a shorter inter-injury interval leads to a more significant acute microglial activation and prolonged astrogliosis in select regions of the brain, compared to the same number of administered over a longer time-period (193). The corpus callosum, hippocampus and lateral septum appear particularly vulnerable to injury (191, 193) and these areas may contribute to clinical symptomology, including anxiety and memory problems (191, 193). Frequent mild head injury also promotes trigeminal sensitivity with associated microglial proliferation and increased neuropeptide levels in the trigeminal pain system (194), which is associated with headaches and migraine that accompany post-concussion syndrome. In a mouse model of highly repetitive mild TBI, 30 injuries cause white matter pathology, and microglial proliferation and activation. This pathology is present 60 days after final injury, and is still apparent at 1 year (195).

### The Immune Response in Animal Models of Mild TBI and Alzheimer's Disease

As discussed above, epidemiological studies associate increased risk of AD-related clinical symptoms with a history of mild TBI (2, 26, 33, 35). To investigate the link between mild TBI and AD, studies have performed brain injury in genetic mouse models of AD and assessed the neuroinflammatory response driving pathology and clinical symptoms (196–200). In APP/PS1 mice that contain human transgenes for both mutated APP and PSEN1 (201), neuroinflammatory gene expression is increased seven days after injury and microglia activation is greater at 2 months in APP/PS1 compared to WT mild TBI controls (197). Importantly, a small molecule inhibitor, previously described to selectively limit pro-inflammatory cytokine production after mild TBI (202), improved cognitive behavior outcomes in APP/PS1 mice after injury (197). Thus, providing a link between neuroinflammatory responses and altered risk for AD-associated pathology changes after mild TBI (197). Other studies in APP/PS1 mice and mild TBI models have investigated acute neuroinflammatory outcomes in young and old mice, however, the interplay between the immune response and AD-like progression was more complicated as neuroinflammation is increased in aged WT mice but reduced in aged APP/PS1 mice (199, 200). In a repeated mild TBI model (12 injuries in 1 month) and aged APP/PS1 mice, there was no increase in various makers of microglia activation 1 month after final brain injury compared to sham control (203). However, there was an increase in brain insoluble to soluble Aß42 ratio in injured APP/PS1 mice compared with sham and a parallel reduction in phagocytic receptor, TREM2, suggesting pathology mediating microglia changes induced by injury (203). In a transgenic mouse model for human tau (hTau), acute microglia activation after mild TBI is increased in both young and aged animals, however, this response is not as robust in aged animals compared to young, suggesting a diminished acute microglial response to mild TBI in animals with established AD pathology (204) and highlights the complicated nature of priming and tolerance in chronic neurodegenerative disease (53).

To model subthreshold injury be expected in competitive contact sports, a 7 days 42-impact paradigm with helmets in mice was used to simulate frequent head injury (196). This paradigm resulted in chronic gliosis and T-cell infiltration of the superior colliculus and optic tract, with concomitant demyelination of the optic nerve and associated white matter tracts 1 month after injury (196). When injuries were performed in Tau58.4 mice, there was progressive neuroinflammation and neurodegeneration in multiple brain regions compared to WT mice. To investigate the role of T-cells in these specific areas vulnerable to demyelination, T-cell-deficient Rag2/ Il2rg (R2G2) mice were subjected to the same injury paradigm. R2G2 mice had altered myeloid cell gene expression and fewer demyelinated lesions compared to T cell-competent mice. This study suggests that vulnerable regions known to be affected in CTE, such as white matter, may be protected by manipulating the immune system (196).

In summary, animal models of mild TBI are varied, but extremely powerful tools investigate mechanisms of injury. There is now a wealth of evidence linking the immune response to mild TBI and the behavioral and pathological outcomes. This is fueling many investigations into new therapeutics targeting these mechanisms.

## Conclusion

Mild TBI, often referred to as concussion, is the most common form of traumatic brain injury and can result in insidious effects including emotional and cognitive dysfunction. In addition, a history of mild TBI is proposed as a risk factor for longer term neurodegenerative disease. Despite this, the underlying pathology is still unclear. Immune activation, and particularly changes to microglia are associated with human mild TBI and a variety of animal models of mild brain injury. In many cases these are descriptive, although evidence suggests immune activation correlates with cognitive and behavioral symptoms. Animal studies are beginning to demonstrate a causative role of the immune system in acute brain dysfunction following mild TBI. The fact that the immune system is readily reactive, and can remain active over a long period of time after injury, leaves open the possibility that an aberrant immune response, driven by factors that have shown to be important for neurodegenerative disease, may contribute to the long-term consequences of mild brain injury.

## Author Contributions

LV contributed to writing the manuscript. HP edited the manuscript. AG wrote and edited the manuscript. All authors contributed to the article and approved the submitted version.

## Conflict of Interest

The authors declare that the research was conducted in the absence of any commercial or financial relationships that could be construed as a potential conflict of interest.
